# Nikshay Mitra: A Remarkable Initiative in Efforts Against Tuberculosis

**DOI:** 10.7759/cureus.62615

**Published:** 2024-06-18

**Authors:** Sankalp Yadav

**Affiliations:** 1 Medicine, Shri Madan Lal Khurana Chest Clinic, New Delhi, IND

**Keywords:** pradhan mantri tb mukt bharat abhiyaan, mtb (mycobacterium tuberculosis), tuberculosis, tb, nikshay mitra

## Abstract

Tuberculosis continues to haunt the fragile healthcare systems in the developing world. It is a disease that is not only limited to the illness due to the bacterial infection but is associated with a number of other impacts, like social and psychological ones. Eliminating tuberculosis is an arduous task and requires a number of initiatives that were taken by the national governments and collaborating partners. One such remarkable development is the introduction of ‘Nikshay Mitra’. It is an initiative where the donors are encouraged to support the tuberculosis patients by providing nutritional, additional diagnostic, and vocational support. Even after nearly two years of introduction, there is a paucity of data, especially from large-scale studies from across India. This editorial sheds light on this initiative.

## Editorial

Tuberculosis is a bacterial infection with an extensive effect on the physical, social, and psychological well-being of patients. This disease is highly prevalent in the endemic countries of Asia, Africa, etc. [1]. Tuberculosis control is part of the targets of Sustainable Development Goals, which aim to end this epidemic by 2030 [2]. Although great success in terms of timely diagnosis, notification, and treatment initiation has been achieved in tuberculosis, the elimination targets appear to be far away. The situation is demanding new and program-centric initiatives to eliminate tuberculosis. In countries like India, which aims to achieve tuberculosis elimination by 2025, there have been several remarkable developments that have happened in the last decade. Some of these include daily directly observed therapy short course, ‘Nikshay Poshan Yojna’, active case-finding campaigns, and use of molecular tools for *Mycobacterium tuberculosis* detection such as the Xpert MTB/RIF (Cepheid, Sunnyvale, CA, USA), recruitment of ‘TB champions’, shorter treatment regimens for drug-resistant tuberculosis, and the inclusion of newer drugs like bedaquiline, pretomanid, and delamanid, etc. In the constant efforts to eliminate the oldest known infectious disease to mankind, another important initiative named ‘Nikshay Mitra’ under ‘Pradhan Mantri TB Mukt Bharat Abhiyan’ was launched in September 2022 [3].

Although the government is making tireless efforts, it is evident that community involvement is necessary for the objective of elimination to be attainable. Hence, the initiative of ‘Nikshay Mitra’, which is aimed at providing support to the patients of tuberculosis at three levels - nutritional, additional diagnostic, and vocational support - is included in the tuberculosis elimination program [3]. The community is sensitized to adopt tuberculosis patients (through multiple information, education, and communication activities), and these donors are called ‘Nikshay Mitra’. Through this program, patients in need are matched with interested donors who agree to donate food, including locally available, culturally appropriate, and nutrient-dense food kits valued at INR 800 per month, until the patient's treatment is over. Staff from the National Tuberculosis Elimination Program must locate the donor and facilitate communication between them and the receiver. The entire initiative program would function through the Nikshay 2.0 portal. These ‘Nikshay Mitras’ can be individuals, non-governmental organizations, cooperative societies, faith-based organizations, cooperates, political parties, and others. For any type of support they choose to provide, a Mitra may adopt a minimum of one consenting tuberculosis (either drug-sensitive or drug-resistant) patient undergoing treatment for a minimum of one year and a maximum of three years. Further, in this initiative, ‘Nikshay Mitras’ could sponsor a locally available, culturally accepted food basket that includes cereals, 3 kg of millet, 1.5 kg of pulses, vegetable oil, groundnuts, and milk. Furthermore, there is a provision to add micronutrients per the recommended daily allowance, like vitamins and mineral supplements [3].

Oftentimes, the patients with tuberculosis are from extremely poor economic backgrounds. Moreover, if the earning member of a family is infected, there is a substantial loss of income, which compromises the financial condition of the whole family. The benefit of these ‘Nikshay Mitras’ is that their assistance could directly reduce the cost of treatment spent by the patients and also improve their nutritional status.

Although the Government of India is disbursing INR 500 per month to tuberculosis patients under the ‘Nikshay Poshan Yojna’, the overall expenses of the patients related to tuberculosis are high. There is a stigma associated with the disease, and there is a prevailing misconception about the disease among patients and society [1]. This has devastating effects on the patients. The initiative of ‘Nikshay Mitra’ is a significant development, as it will bring communities and patients together. This would eventually result in reducing the psychological and social impact of the disease. Additionally, this initiative would also result in the dissemination of information about tuberculosis awareness, diagnosis, and management. The vocational support would help patients earn their livelihood in cases of job loss [3].

However, this initiative is still in its early stages, and the published data regarding the effects on patients and society is sparse. Data from some parts of India indicates a remarkable improvement in treatment adherence and success rates (reaching 94% for a cohort of 304 tuberculosis patients with no fatalities and no loss to follow-up cases) [4]. While some other studies have reported challenges associated with this initiative like the disease-related stigma, privacy concerns, donor retention issues, supply disruptions, and insufficient quantities of food items [5].

To conclude, newer initiatives like ‘Nikshay Mitra’ are a welcome addition to boost the efforts aimed at tuberculosis elimination. These initiatives could bridge the gap between society and tuberculosis patients. However, with the paucity of data available on this initiative, it is imperative that large-scale studies from various parts of the country be done to provide concrete evidence for extending the reach of such initiatives, and this would also highlight the ground-level challenges associated with this novel scheme.
